# Effect of Formulated Edible Oils From Groundnut and African Walnut Oils on Some Hematological, Inflammation, and Oxidative Stress Markers in High‐Fat Diet‐Induced Obese Wistar Rats

**DOI:** 10.1002/fsn3.70130

**Published:** 2025-04-17

**Authors:** Thelma Besong Taku, Fabrice Tonfack Djikeng, Bertrand Ayuk Tambe, Veshe‐Teh Zemoh Sylvia Ninying, Evans Mainsah Ngandung

**Affiliations:** ^1^ School of Home Economics, Tourism and Hotel Management Chartered Higher Institute of Technology and Management Buea Cameroon; ^2^ Department of Biochemistry and Molecular Biology, Faculty of Science University of Buea Buea Cameroon; ^3^ Department of Public Health and Hygiene, Faculty of Health Science University of Buea Buea Cameroon; ^4^ School of Health Science, Department of Public Health and Administration, Nutrition and Dietetics Biaka University Institute of Buea Buea Cameroon; ^5^ Department of Chemistry, Faculty of Science University of Buea Buea Cameroon

**Keywords:** formulated edible oils, hematology, inflammation, obesity, oxidative stress

## Abstract

The objective of this study was to evaluate the effects of groundnut oil, African walnut oil, and their blends on some biochemical parameters in obese Wistar rats. Obesity was induced with a high‐fat diet for 60 days and managed with oils and orlistat for 28 days. The rats were sacrificed on the 29th day, and the serum and blood were collected. The serum was used to evaluate oxidative stress and cytokine markers, while the blood was used for hematology studies. Results showed that oil quality indices were within standard ranges as recommended by the norm. Hematological assessments showed no significant differences in most parameters across groups, except for platelet counts, which were lower in the group taking 100% of groundnut oil. Catalase activity and glutathione peroxidase (GSH) levels were evaluated in various organs. The normal group exhibited the highest catalase activity in the brain and liver compared with the rats that received the high‐fat diet. Notably, GSH activity was higher in the brains of rats receiving the 60:40 blend. Thiobarbituric acid (TBA) values indicated lower oxidative stress in the normal and 100% walnut oil groups. Nitric oxide concentrations were significantly higher in the normal and walnut oil groups compared with the negative control, suggesting a protective effect against oxidative stress. Cytokine analysis revealed elevated inflammatory markers in the negative control group, highlighting the potential anti‐inflammatory properties of the oils. These findings suggest that groundnut oil, African walnut oil, and their blends might have anti‐inflammatory activities, might preserve hematological markers, and protect against oxidative stress.

## Graphical abstract

Groundnut oil, African walnut oil, and their 50:50 and 60:40 blends have anti‐inflammatory properties in high‐fat diet–induced Wistar rats. Groundnut oil, African walnut oil, and their 50:50 and 60:40 blends have antioxidant properties in high‐fat diet induced in Wistar rats. Groundnut oil, African walnut oil, and their 50:50 and 60:40 blends preserve the hematological markers in high‐fat diet–induced Wistar rats.

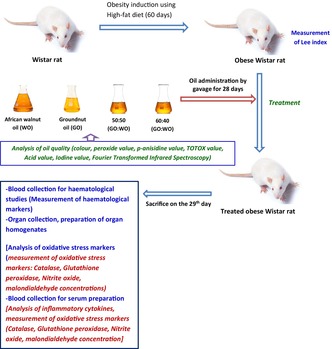

## 1 Introduction

Obesity being a leading cause of many other diseases such as cardiovascular diseases, hypertonia, and diabetes has become a topic of interest over the years and even in present times (Thomas et al. 2020). It is stipulated that by 2025, about 18% of men and 21% of women will be obese worldwide (Kilen et al. 2018). In 2014, an estimated number of about 600 million people worldwide were obese compared with over 105 million of the world's population being obese in 1975 (Ji et al. 2017). This explains why in some countries, obesity is considered a pandemic and a serious public health problem due to its high prevalence (Swinburn et al. 2011).

Dietary habits, genetic, and epigenetic factors are some of the causes of obesity. These dietary habits, as described by the Global Nutrition Report (2016) are characterized by high fat, carbohydrate, and calorie diets (Ji et al. 2017). Some studies showed evidence that obesity has a positive relationship with the lipid composition of the diet, causing an excessive fat accumulation imbalance in the body that is not expended (Marques‐Lopes et al. 2004).

Unhealthy diets, especially those rich in fatty acids and sugars, are the most behavioral risk factors of obesity and its complications (WHO 2019). Western diets are associated with this risk as they contain large amounts of saturated fatty acids (Simopoulos 2008; Martin et al. 2006). As a result, cardiovascular diseases, which are one complication of obesity, have surpassed cancer as the top cause of death worldwide, killing more than a million people each year, with one‐third of the deaths occurring before age 70 (WHO 2017). Unhealthy diets, lack of physical activity, heavy drinking, and smoking are associated with factors causing these non‐communicable diseases (Dzudie et al. 2020).

Moreover, dietary monounsaturated (MUFAs) and polyunsaturated fats (PUFAs) are proposed to have correlated effects with the reduction of obesity, hypertension, CVDs (cardiovascular diseases), diabetes mellitus, coronary heart disease, etc. (Lands 2012; Yehuda and Rabinovitz 2016). In Cameroon, palm oil and its liquid fraction, palm olein, which are rich in saturated fatty acids (about 50%) are the most popular and most widely used oils in households. They are used almost every day in the preparation of meals. This is because of the lack of funds to purchase oils rich in polyunsaturated fatty acids such as soybean oil, cottonseed oil, sunflower oil, etc. On the other hand, the consumption of fast foods, which are rich in saturated fatty acids and sugar, keeps increasing. Embracing a diet rich in polyunsaturated fatty acids can help reduce the risk of obesity, coronary heart diseases, hypertension, etc. (Mensah 2008; De Souza et al. 2015).

Previous studies reported the role of polyunsaturated fatty acids in the management or prevention of non‐communicable diseases, among which are obesity (Imamura et al. 2016). Most of these studies referred to single oils either rich in MUFAs or PUFAs. The efficiency of single oils as functional foods compared with multisource edible oils has been reported to be lower, since oil mixtures are suspected to be more balanced in nutraceutical compounds such as omega‐3 and omega‐6 fatty acids (Rifna et al. 2022). The efficiency of some multisource edible oils on some markers of non‐communicable diseases has been reported (Hilton et al. 2019). It will be important to formulate such oil from the oils locally available in Cameroon and evaluate their beneficial effects on some markers of non‐communicable diseases.

Due to recent urbanization and cultivation of Western cultures, Cameroon has been on the rising wave of unhealthy dietary habits, a low level of physical activity, and a sedentary lifestyle which are one of the fueling causes of obesity and other non‐communicable diseases (Echouffo‐Tchegui and Kegne 2011). According to FAO (2019), obesity in Cameroon's adult population increased from about 4.9% in the year 2000 to 9.5% in 2016. A report from Fezeu et al. (2008) highlights the fact that the obesity pandemic is increasingly taking an important place as a cause of disease burden in Cameroon and this may escalate if no action is taken (Simo et al. 2020). Despite the expensive nature of the anti‐obesity treatments such as orlistat, statins, and leptin (McDuffie et al. 2021), obesity still remains at pandemic levels (Baker et al. 2022). However, recent interventions on obesity management include more natural ways such as the use of nutraceuticals, dietary supplements, and functional foods which have been proven to prevent or manage the disease and its complications (Vishvakarma et al. 2023; Biesalski 2001). Among these functional foods or ingredients are edible oils. There is very limited knowledge of multisource edible oils in Cameroon. Plant sources of omega‐3 fatty acids are very rare, since oils such as soybean and cottonseed oils, which are the most common polyunsaturated oils in the country, contain a good amount of omega‐6 fatty acids but only a few omega‐3. Identifying sources of omega‐3 and making blends with WHO ratios of omega‐6 and omega‐3 will be of great importance in preventing obesity and its complications in Cameroon. Among the plant sources of omega‐3 in Cameroon is African walnut oil, which the seeds are highly available between July and September of each year but remain underexploited for their oil and are instead boiled and consumed. Its oil was proven to contain 70.39% of omega‐6 and 14.41% of omega‐3 fatty acids (Tchiegang et al. 2007). Mixing this omega‐3–rich oil with groundnut oil (GO), which is well known in Cameroon, and testing their effects on some markers of obesity and their complications can be of good interest for the population. There is therefore a need to evaluate the effects of multi‐source edible oils from groundnut and African walnut oils on some hematological, inflammation, and oxidative stress markers in high‐fat diet (HFD)‐induced obese Wistar rats. It can be hypothesized that multi‐source edible oils from groundnut and walnut oils significantly improve some markers of obesity and its complications in HFD‐induced obese Wistar rats. Thus, the objective of this study was to evaluate the effects of multi‐source edible oils from groundnut and African walnut oils on some markers of obesity and its complications in HFD‐induced obese Wistar rats.

## 2 Materials and Methods

### 2.1 Materials

Fresh African walnuts were purchased from a farmer in Muyuka, Fako Division, South‐West Region of Cameroon in October 2023. Groundnut was bought from the market in the Buea municipality in October 2023. Forty‐two albino Wistar rats aged 3–4 months and weighing 150–200 g were purchased from a farmer in Yaoundé, Center Region of Cameroon. All reagents and chemicals used were of analytical grade.

### 2.2 Methods

#### 2.2.1 Extraction, Degumming, and Partial Purification of Groundnut and African Walnut Oils

##### 2.2.1.1 Oils Extraction

African walnuts were cracked to remove the shell and the nuts were cut into small pieces and dried in an electric air‐dried oven at 50°C for 24 h. The dried nuts were cold‐pressed to obtain the oil. About two kilograms of African walnuts was used. The extracted oil was collected in a beaker gradually and later stored in the freezer at −18°C for further analysis and the defatted cake was discarded. GO was extracted following the same method.

##### 2.2.1.2 Degumming

The degumming process was done according to the method described by Moretto and Fett (2014) with slight modifications. In a beaker of 250 mL, 100 g of oil and 3 mL of warm water (70°C) were added. The mixture was stirred for 30 min, and allowed to decant. The precipitate was removed and the oil was filtered using anhydrous sodium sulfate.

##### 2.2.1.3 Partial Purification

Purification of oil samples was done using palm kernel shell activated carbon and decolorized activated charcoal at 1.5% each (Ulfah et al. 2023). Purification was done by adding 100 mL of oil into a 250 mL Erlenmeyer. After that, activated carbon was added (1.5%). The Erlenmeyer containing the oil and activated carbon was coated with aluminum foil, heated, and stirred on a hot plate magnetic stirrer for 60 min at 70°C. A vacuum filter was used to isolate the filtrate. The filtrate was transferred in bottles coated with aluminum foil and kept in the freezer at −18°C for further analysis.

#### 2.2.2 Formulation of Multisource Edible Oils

Multisource edible oils from groundnut and African walnut were prepared in two different ratios, 50:50 and 60:40 as recommended by the Food safety and Standards Authority of India FSSAI (2021). The uniform multisource oils were prepared by stirring the sample at 180 rpm for 15 min as reported by Dhyani et al. (2022). It is important to note that saturated oils were warmed at their melting temperatures before use. The samples as well as their blends that were used are presented in Table 1.

**TABLE 1:** Composition of different oil samples.

Samples	Composition
1	African walnut oil (100%)
2	Groundnut oil (100%)
3	Groundnut oil (50%) + African walnut oil (50%)
4	Groundnut oil (60%) + African walnut oil (40%)

The above‐mentioned oils were analyzed for their initial quality. The parameters analyzed were the color, peroxide, *p*‐Anisidine, TOTOX, acid values, and the Fourier‐transformed infrared spectroscopy. The data of these analyses are available in our previous publication (Douky et al. 2025).

#### 2.2.3 Animal Bioassay

##### 2.2.3.1 Ethical Clearance

Animals were cared for and used in agreement with international standard guidelines for animal use. To carry out this study, an ethical clearance for animal handling and care was obtained from the University of Buea—Institutional Animal Care and Use Committee (IACUC) with permit UB‐IACUC No 02/2024.

##### 2.2.3.2 Preparation of Animal Feed

The method described by Othman et al. (2019) was used in the formulation of the normal and HFDs with slight modifications. The normal diet included the following ingredients: 600 g of maize flour, 200 g of soybeans, 290 g of fish powder, and 10 g of salt for each kilogram of food. The HFD consisted of 600 g of normal diet, 110 g of boiled egg yolk as a source of cholesterol, and 290 g of lard. These proportions were for one kilogram of food. After a dough‐like consistency was formed using water, the feed was shaped into small balls and dried in the oven at 40°C overnight and used to feed the rats the next morning. Feed was prepared every day to avoid chemical and biological spoilage.

##### 2.2.3.3 Treatment of Animals

Upon collection of the rats, they were allowed to acclimatize for a 12 h light–dark cycle at room temperature for 14 days in cages containing sawdust and were given water and food ad libitum before the start of the experiment. A total of 42 adult male rats were randomly distributed into seven groups of six rats each. Groups 2, 3, 4, 5, 6, and 7 were fed the HFD for 60 days, and the oils and Orlistat were administered to the rats of groups 3, 4, 5, 6, and 7 for an additional 28 days. The characteristic of each group is presented in Table 2.

**TABLE 2:** Animal group distribution and characteristics.

Groups	Characteristics
Group 1 (normal)	Healthy rats fed with the normal rodent chow
Group 2 (negative control)	Obese rats + distilled water (250 mg/kg bodyweight (BW))
Group 3 (positive control)	Obese rats + oral administration of Orlistat (10 mg/kg) for 28 days
Group 4 (test group 1)	Obese rats + oral administration of African walnut oil (1000 mg/kg) for 28 days by gavaging
Group 5 (test group 2)	Obese rats + oral administration of groundnut oil (1000 mg/kg) for 28 days by gavaging
Group 6 (test group 3)	Obese rats + oral administration of 1000 mg/kg of a 50:50 (GO:WO) multisource edible oil from groundnut and African walnut oils for 28 days by gavaging
Group 7 (test group 4)	Obese rats + oral administration of 1000 mg/kg of a 60:40 (GO:WO) multisource edible oil from groundnut and African walnut oils for 28 days by gavaging

After 28 days of treatment, the rats were fasted overnight and anesthetized using ketamine 60 mg/kg and diazepam 10 mg/kg body weight and the blood was collected by cardiac puncture using a 5 mL syringe. The blood was divided into two portions; the first one was introduced in tubes with EDTA and was used for hematological evaluation. The second was introduced in a tube without EDTA and the serum was obtained by centrifugation (3000 rpm for 15 min). The serum was used for the evaluation of oxidative stress and inflammatory cytokine markers. Organs of interest (heart, kidney, pancreas, brain, spleen, and liver) were collected and weighed. They were used to prepare organ homogenates (22 g of organ/100 mL of distilled water) which were centrifuged at 3000 rpm for 10 min and used for the determination of oxidative stress markers.

##### 2.2.3.4 Determination of Hematological Parameters

Hematological analyses were carried out on blood samples introduced in tubes containing EDTA. They were done using an automatic hematological analyzer (SFRI H18 LIGHT auto Hematology Analyzer). The parameters analyzed were white blood cells (WBC), red blood cells (RBC), hemoglobin (HGC), hematocrit (HCT), mean corpuscular volume (MCV), granulocytes (GRAN), platelet (PLT), mean platelet volume (MPV), platelet large cell ratio (PLCR), platelet distribution width (PDW), mean corpuscular hemoglobin (MCH), and MCHC.

##### 2.2.3.5 Analysis of Some Inflammatory Cytokines

Serum was used for the analysis of inflammatory cytokines. The sera were used for ELISA testing (Rat TNF‐α, Duo Set, Cat no: DY510‐05, R&D systems), (Rat INF‐γ, Duo Set, Cat no: RIF00, R&D systems), (Rat IL‐1β, Duo Set, Cat no: RLB00‐1, R&D systems), and (Rat IL‐6, Duo Set, Cat no: DY506‐05, R&D systems) for TNF‐α, INF‐γ, IL‐1β, and IL‐6 concentrations, respectively, as described by the manufacturer. Rat antibodies for ELISA tests were specific for each cytokine determined in the sample, respectively.

##### 2.2.3.6 Determination of Oxidative Stress Parameters

Serum and organ homogenates were used for the measurement of oxidative stress parameters. Malondialdehyde (MDA) level was measured following the method described by Wilbur (1990). The reduced glutathione (GSH) was assayed as described by Ellman (1959); the catalase activity was determined following the method reported by Sinha (1972) and the nitric oxide (NO) level was determined following the method reported by Montgomery and Doymock (1961).

### 2.3 Statistical Analysis

The obtained data (mean ± standard deviation) were subjected to one‐way analysis of variance (ANOVA) using Statgraphics Centurion version XVI to evaluate the statistical significance of the data. A probability value at *p* < 0.05 was used for statistical significance.

## 3 Results

### 3.1 Oil Quality

**FIGURE 1:**
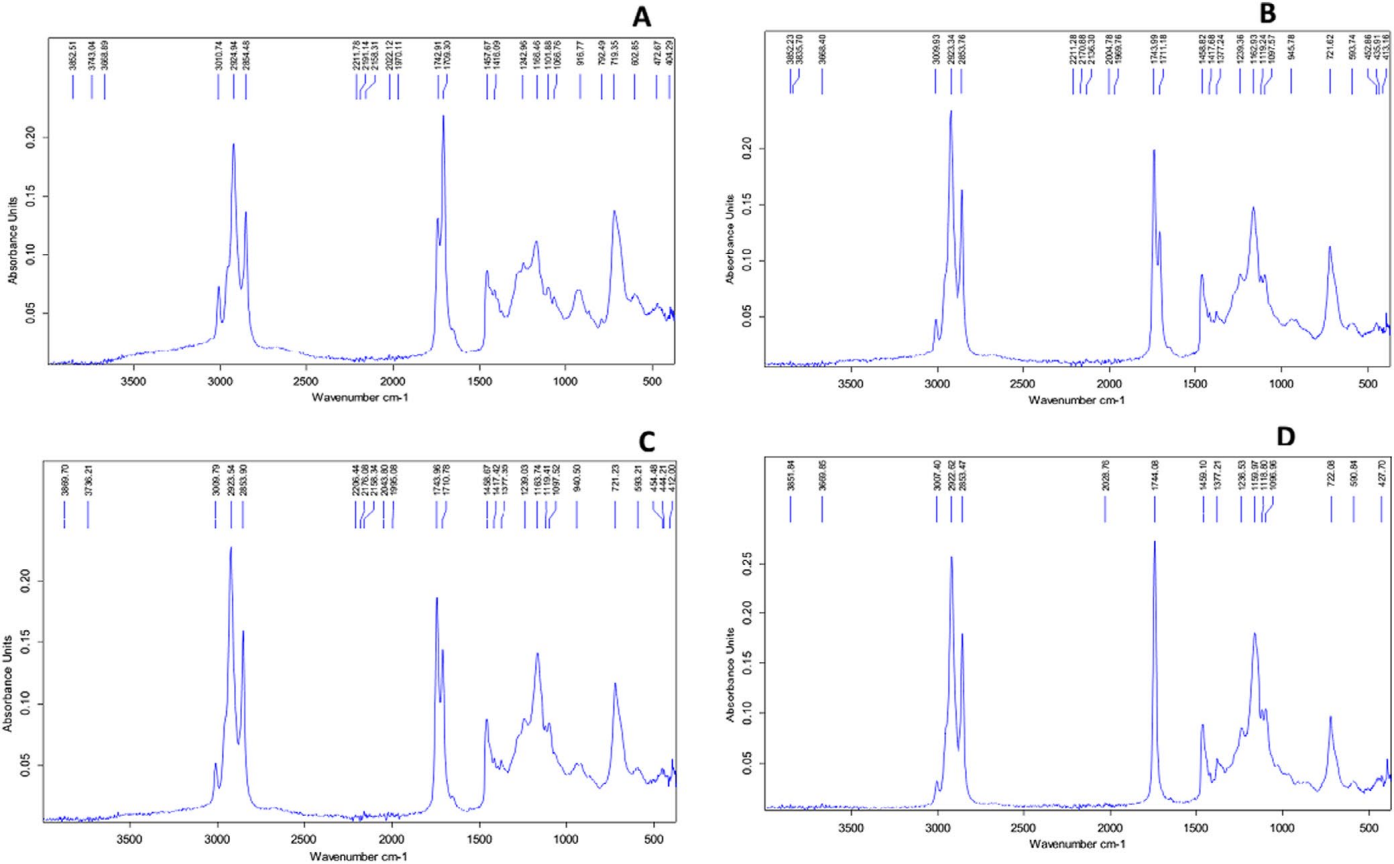
Fatty acid chromatogram of A = Groundnut oil; B = African walnut oil; C = 50:50 (Groundnut oil:African walnut oil); D = 60:40 (Groundnut oil:African walnut oil) (*Source*: Douky et al. 2025). *N* = 6. Values are presented as mean ± SD. ^a–d^values of the same parameters with different superscripts are significantly different at *p* < 0.05.

The results presenting the initial characteristics of oil samples are presented in Table 3, which are the data we previously published in a similar study but on other biochemical parameters using the same oil samples (Douky et al. 2025). The Fourier‐transformed infrared spectrum of the same oil samples was also done in our previous study, and the result is presented in Figure 1 (Douky et al. 2025).

**TABLE 3:** Initial physicochemical properties of formulated multisource oil samples.

Parameter	Groundnut oil (GO) (100%)	Walnut oil (WO) (100%)	50:50 (GO:WO)	60:40 (GO:WO)	Standard (WHO/FAO 1999, 2017)
Color	*L* −14.57 ± 0.00^a^ More black	***L*** −27.15 ± 0.94^b^ More black	*L* −14.44 ± 0.03^a^ More black	*L* −14.50 ± 0.07^a^ More black	/
	a* −0.96 ± 0.01^a^ Green	a* −0.58 ± 0.00^b^ Normal	a* −0.78 ± 0.03^c^ Green	a* −0.82 ± 0.00^c^ Green	/
	b* −0.53 ± 0.04^a^ Blue	b* 4.52 ± 0.82^b^ More yellow	b* 0.99 ± 0.09^c^ Yellow	b* 0.77 ± 0.03^c^ Yellow	/
Peroxide value (meq O_2_/kg)	4.01 ± 0.39^a^	2.52 ± 0.00^b^	4.94 ± 0.03^a^	4.93 ± 0.00^a^	≤ 15 meq O_2_/kg
*p*‐Anisidine value	7.09 ± 0.16^a^	12.33 ± 0.00^b^	10.75 ± 0.08^b^	11.81 ± 0.07^b^	≤ 20 in fish oil
TOTOX value	17.26 ± 2.65^a,c^	16.83 ± 0.00^a^	20.63 ± 0.56^c^	21.68 ± 0.07^c^	≤ 26 in fish oil
Acid value (mg KOH/g)	0.13 ± 0.02^a^	20.34 ± 0.22^b^	10.81 ± 0.00^c^	8.73 ± 0.67^c^	≤ 24 mg KOH/g
Iodine value (g I_2_/100 g)	86.41 ± 2.32^a^	166.41 ± 3.54^b^	112.12 ± 4.32^c^	93.21 ± 2.33^c^	/

*Note:* *n* = 3 values are presented as mean ± SD. Different letters in a row indicate significant difference between samples (*p* < 0.05).

Abbreviations: GO, Groundnut oil; 50:50 (GO:WO), Groundnut oil: African walnut oil in the ratio 50:50; 60:40, Groundnut oil: African walnut oil in the ratio 60:40; WO, African walnut oil.

*Source:* Douky et al. (2025).

### 3.2 Hematological Parameters

Table 4 presents the hematological parameters of animals. No significant (*p* > 0.05) difference was recorded in the WBC, RBC, HGC, MCV, MCH, MCHC, MPV, PCT, P‐LCR, and PDW concentrations of all groups. The group treated with 100% GO exhibited the lowest (*p* < 0.05) PLT. The normal group presented the highest (*p* < 0.05) HCT value compared with the other groups. Concerning the GRAN, they were higher in the blood of rats treated with 100% GO and GO:WO (60:40) compared with the other groups. However, no significant (*p* > 0.05) difference was observed in the GRAN value of these other groups. At the level of LYM, the group treated with GO:WO (60:40) presented the lowest (*p* < 0.05) concentration compared with the other groups. The MID of rats treated with oil blends was significantly (*p* < 0.05) higher than those of the other groups.

**TABLE 4:** Effect of African walnut oil, groundnut oil, and their blends on hematological parameters in HFD‐induced obese Wistar rats.

	WBC	LYM	MID	GRAN	RBC	HGC	HCT	MCV	MCH	MCHC	PLT	MPV	PCT	P‐LCR	PDW
Normal	2.90 ± 0.72^a^	66.00 ± 3.45^ab^	16.87 ± 1.25^a^	18.46 ± 2.67^a^	7.29 ± 0.82^a^	14.36 ± 2.08^a^	54.86 ± 1.36^a^	54.86 ± 1.22^a^	17.86 ± 1.72^a^	32.64 ± 0.00^a^	829.66 ± 104.88^ab^	6.48 ± 0.23^a^	0.546 ± 0.08^a^	8.27 ± 1.03^a^	8.42 ± 0.26^a^
Negative control	3.00 ± 0.14^a^	61.46 ± 7.01^ab^	19.53 ± 1.24^ab^	22.10 ± 3.25^a^	7.51 ± 1.16^a^	14.07 ± 1.60^a^	43.86 ± 4.09^b^	56.35 ± 2.16^a^	17.52 ± 1.06^a^	31.84 ± 3.35^a^	734.50 ± 52.84^ab^	6.04 ± 0.30^a^	0.42 ± 0.18^a^	5.66 ± 1.12^a^	7.84 ± 0.99^a^
Orlistat (10 mg/kg)	5.46 ± 1.49^a^	66.95 ± 7.24^ab^	18.72 ± 2.29^ab^	21.36 ± 0.55^a^	7.65 ± 0.44^a^	13.16 ± 0.98^a^	39.50 ± 2.48^b^	51.70 ± 1.52^a^	17.15 ± 0.70^a^	33.25 ± 0.68^a^	774.00 ± 78.31^ab^	6.31 ± 0.24^a^	0.48 ± 0.05^a^	8.15 ± 1.96^a^	8.46 ± 0.58^a^
100% WO (1000 mg/kg)	5.32 ± 1.10^a^	61.90 ± 4.71^ab^	23.67 ± 3.21^b^	20.86 ± 3.20^a^	7.82 ± 0.77^a^	13.65 ± 1.61^a^	41.22 ± 3.96^b^	52.60 ± 1.56^a^	16.63 ± 1.34^a^	31.73 ± 2.13^a^	679.00 ± 0.00^b^	6.30 ± 0.04^a^	0.04 ± 0.00^a^	7.52 ± 0.70^a^	8.21 ± 0.93^a^
100% GO (1000 mg/kg)	5.76 ± 0.55^a^	68.13 ± 10.01^bc^	17.75 ± 4.78^ab^	32.43 ± 2.43^b^	7.73 ± 0.53^a^	13.46 ± 2.10^a^	40.86 ± 4.00^b^	52.73 ± 1.70^a^	17.23 ± 1.07^a^	32.73 ± 1.40^a^	576.00 ± 27.20^c^	6.21 ± 0.24^a^	0.39 ± 0.07^a^	6.83 ± 2.19^a^	7.85 ± 0.18^a^
50:50 (GO: WO) (1000 mg/kg)	5.52 ± 0.77^a^	55.22 ± 3.04^bc^	21.56 ± 1.27^b^	20.1 ± 0.10^a^	7.79 ± 0.45^a^	13.68 ± 0.95^a^	40.94 ± 2.16^b^	52.52 ± 0.95^a^	17.40 ± 0.52^a^	33.18 ± 0.68^a^	664.33 ± 16.82^b^	6.10 ± 0.20^a^	0.39 ± 0.02^a^	5.62 ± 0.20^a^	7.94 ± 0.3^a^
60:40 (GO: WO) (1000 mg/kg)	6.32 ± 3.32^a^	46.12 ± 5.32^c^	24.95 ± 2.40^b^	32.76 ± 2.76^b^	7.78 ± 0.43^a^	14.8 ± 0.35^a^	41.28 ± 0.88^b^	52.37 ± 1.81^a^	17.07 ± 0.48^a^	32.70 ± 0.47^a^	629.66 ± 70.01^b^	6.20 ± 0.31^a^	0.39 ± 0.04^a^	7.63 ± 2.08^a^	8.15 ± 0.51^a^

*Note:* *n* = 6. Values are presented as mean ± SD. ^a–c^Values of the same column with different superscripts are significantly different at *p* < 0.05.

### 3.3 Inflammatory Cytokines

**FIGURE 2:**
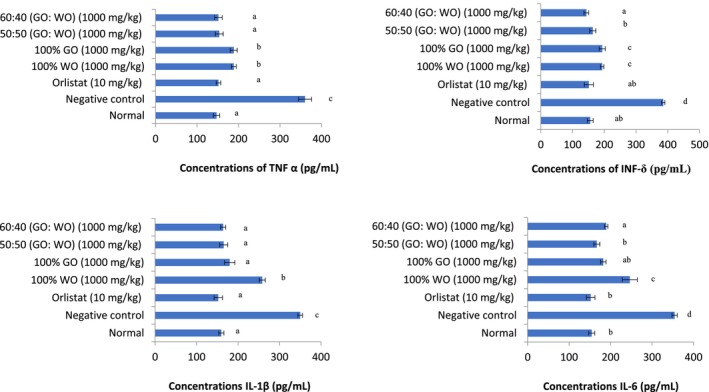
Inflammatory cytokines (TNF‐α, INF‐δ, IL‐1β, and IL‐6) of animal sera.

Figure 2A–D presents the inflammatory cytokines (TNF‐α, INF‐δ, IL‐1β, and IL‐6) of the sera of animals. Generally, the negative control group presented significantly (*p* < 0.05) higher cytokine levels compared with the other groups.

### 3.4 Effect on Oxidative Stress Parameters

#### 3.4.1 Effect of Oils on CAT Activity in Organ Homogenate and Serum

The catalase activity in organ homogenate and sera is presented in Table 5. No significant (*p* > 0.05) difference was observed in the catalase activity of all rat groups at the level of the spleen, kidney, and heart. At the level of the brain, the normal group, the negative control group, and the group treated with orlistat presented similar (*p* > 0.05) catalase activities. However, they were significantly (*p* < 0.05) lower than that of the other groups. At the level of the pancreas, the catalase activity of rats fed with HFD was generally significantly (*p* < 0.05) lower than that of the normal group. Looking at the liver, the negative control group and the group that received 50:50 (GO:WO) presented the highest catalase activities compared with the other groups. At the level of the serum, 100% WO presented the lowest (*p* < 0.05) catalase activity compared with the other groups.

**TABLE 5:** Effect of African walnut oil, groundnut oil, and their corresponding blends on CAT levels in organ homogenate and serum (μmol H_2_O_2_/min/mg).

	Spleen	Kidney	Heart	Brain	Pancreas	Liver	Serum
Normal	10.92 ± 1.10^a^	11.49 ± 0.46^a^	15.12 ± 1.01^a^	18.48 ± 0.00^a^	14.10 ± 0.00^a^	17.16 ± 0.00^a^	20.37 ± 2.50^a^
Negative control	10.80 ± 0.00^a^	9.90 ± 0.42^a^	14.70 ± 1.78^a^	16.56 ± 4.32^a^	12.69 ± 1.90^ab^	20.01 ± 0.04^b^	18.92 ± 1.41^a^
Orlistat (10 mg/kg)	9.82 ± 0.24^a^	11.08 ± 2.06^a^	15.86 ± 1.17^a^	17.62 ± 1.86^a^	10.20 ± 0.33^b^	17.19 ± 3.86^abc^	20.72 ± 1.78^a^
100% WO (1000 mg/kg)	12.62 ± 1.02^a^	11.67 ± 1.14^a^	13.00 ± 0.29^a^	21.66 ± 1.27^b^	11.32 ± 1.05^b^	15.09 ± 1.82^ac^	14.32 ± 2.43^b^
100% GO (1000 mg/kg)	10.62 ± 0.76^a^	10.66 ± 0.87^a^	14.58 ± 1.61^a^	21.30 ± 2.63^b^	12.39 ± 0.72^ab^	13.02 ± 0.00^c^	26.60 ± 4.70^a^
50: 50 (GO: WO) (1000 mg/kg)	9.72 ± 0.67^a^	10.72 ± 1.80^a^	13.17 ± 0.29^a^	21.00 ± 1.18^b^	10.52 ± 1.30^b^	21.36 ± 0.00^b^	18.60 ± 1.81^a^
60: 40 (GO: WO) (1000 mg/kg)	12.27 ± 3.35^a^	12.26 ± 3.99^a^	15.88 ± 2.41^a^	23.34 ± 3.47^b^	10.47 ± 1.56^b^	15.36 ± 3.82^ac^	24.26 ± 4.54^a^

*Note:* *n* = 6. Values are presented as mean ± SD. ^a–c^Values of the same column with different superscripts are significantly different at *p* < 0.05.

#### 3.4.2 Effect of Oils on GSH Activity in Organ Homogenate and Serum

Table 6 presents the glutathione peroxidase (GSH) activity in organ homogenate and sera of rats. At the level of the spleen, the negative control presented a significantly (*p* < 0.05) higher GSH concentration compared with the other groups. Looking at the kidney, no significant (*p* > 0.05) difference was observed in the GSH activity of all rats, except for the rats that took 100% GO, who presented the lowest (*p* < 0.05) activity. No significant (*p* > 0.05) difference was recorded in the GSH activity of heart homogenates. At the level of the brain, the rats that received 60:40 (GO: WO) 1000 mg/kg presented the highest (*p* < 0.05) GSH activity compared with the other groups. They were followed by rats that consumed 100% WO and the negative control group. Looking at the pancreas homogenate, the normal group and the group that received 100% GO exhibited the highest GSH activities compared with all the other groups. At the level of the liver, rats treated with orlistat presented significantly (*p* < 0.05) higher GSH activity compared with all other groups. The lowest activity was recorded with rats that received 50:50 (GO:WO). The GSH activity in the serum showed that rats that received 50:50 (GO:WO) presented the highest GSH activities. It was followed by the group that received orlistat and the oil blends.

**TABLE 6:** Effect of African walnut oil, groundnut oil, and their corresponding blends on GSH levels in organ homogenate and serum (μmol).

	Spleen	Kidney	Heart	Brain	Pancreas	Liver	Serum
Normal	40.07 ± 0.00^a^	117.50 ± 25.37^a^	142.27 ± 7.27^a^	64.33 ± 0.00^a^	60.07 ± 0.00^a^	129.08 ± 0.67^a^	45.58 ± 0.51^a^
Negative control	126.02 ± 0.00^b^	112.31 ± 18.04^a^	134.55 ± 12.16^a^	104.55 ± 0.00^b^	27.94 ± 14.14^ba^	132.79 ± 0.00^a^	67.94 ± 10.08^b^
Orlistat (10 mg/kg)	24.41 ± 0.00^c^	110.77 ± 2.85^a^	141.37 ± 3.63^a^	75.18 ± 23.24^a^	37.23 ± 18.54^bc^	139.70 ± 1.08^b^	97.53 ± 10.76^c^
100% WO (1000 mg/kg)	62.38 ± 10.24^de^	112.67 ± 20.67^a^	124.60 ± 20.87^a^	115.51 ± 6.23^bc^	21.94 ± 8.57^b^	127.89 ± 17.46^a^	59.74 ± 1.81^bd^
100% GO (1000 mg/kg)	57.13 ± 0.00^d^	72.42 ± 1.76^b^	113.52 ± 10.67^a^	92.96 ± 10.87^ab^	41.86 ± 9.40^c^	125.36 ± 4.37^a^	103.57 ± 6.07^c^
50:50 (GO: WO) (1000 mg/kg)	35.47 ± 6.18^a^	103.25 ± 10.81^a^	107.32 ± 11.59^a^	69.87 ± 10.21^ab^	15.40 ± 1.31^d^	101.20 ± 8.33^c^	62.99 ± 4.01^b^
60:40 (GO: WO) (1000 mg/kg)	73.45 ± 3.74^e^	110.73 ± 9.67^a^	131.17 ± 26.55^a^	120.80 ± 6.23^c^	23.16 ± 0.72^b^	119.28 ± 15.66^a^	87.10 ± 8.15^d^

*Note:* *n* = 6. Values are presented as mean ± SD. ^a–e^Values of the same column with different superscripts are significantly different at *p* < 0.05.

#### 3.4.3 Effect of Oils on MDA Levels in Organ Homogenate and Serum

The thiobarbituric acid (TBA) value of organ homogenates and sera of animals is presented in Table 7. The group receiving the normal diet and 100% WO presented the lowest (*p* < 0.05) TBA values compared with the other groups. At the level of the kidney, the negative control group exhibited a significantly (*p* < 0.05) higher TBA value compared with the other groups. The TBA value in the heart was significantly (*p* < 0.05) lower in the normal group compared with the groups that received the HFD. Generally, no significant (*p* < 0.05) change was recorded in the TBA value of the brain of all rats, except for the groups that received orlistat, which presented the lowest value (*p* < 0.05). At the level of the pancreas, the negative control and the rats treated with orlistat, 100% GO, and 50:50 (GO;WO) presented the highest (*p* < 0.05) TBA values compared with the normal group and the group treated with 60:40 (GO:WO). The lowest values were obtained with the normal group and the group that received 100% WO. The TBA values of the liver were significantly (*p* < 0.05) elevated in the negative control groups and the group fed with 100% GO. At the level of the serum, the highest (*p* < 0.05) value was observed in the negative control.

**TABLE 7:** Effect of African walnut oil, groundnut oil, and their corresponding blends on MDA levels in organ homogenate and serum (μmol/L).

	Spleen	Kidney	Heart	Brain	Pancreas	Liver	Serum
Normal	1.88 ± 0.69^a^	1.04 ± 0.26^a^	1.83 ± 0.02^a^	2.56 ± 0.60^a^	1.56 ± 0.00^a^	1.03 ± 0.07^a^	1.86 ± 0.44^c^
Negative control	4.22 ± 0.00^b^	3.27 ± 0.00^bc^	2.59 ± 0.47^bc^	2.51 ± 0.00^bc^	4.59 ± 0.00^bc^	2.74 ± 0.30^b^	3.01 ± 0.39^b^
Orlistat (10 mg/kg)	3.07 ± 0.79^bc^	1.40 ± 0.00^a^	3.91 ± 0.79^b^	1.98 ± 0.02^b^	3.20 ± 0.04^b^	1.16 ± 0.00^a^	2.35 ± 0.52^bc^
100% WO (1000 mg/kg)	2.08 ± 0.97^c^	2.22 ± 0.07^c^	4.00 ± 0.42^b^	2.25 ± 0.21^a^	1.74 ± 0.11^a^	1.21 ± 0.00^a^	1.85 ± 0.61^ac^
100% GO (1000 mg/kg)	4.71 ± 0.85^b^	1.13 ± 0.23^a^	3.07 ± 0.00^b^	2.23 ± 0.14^a^	3.41 ± 0.65^bc^	2.46 ± 0.36^b^	1.68 ± 0.37^a^
50:50 (GO: WO) (1000 mg/kg)	2.71 ± 0.00^bc^	0.97 ± 0.00^a^	3.34 ± 0.00^b^	2.12 ± 0.00^a^	3.76 ± 0.00^b^	1.32 ± 0.00^a^	2.28 ± 0.33^c^
60:40 (GO: WO) (1000 mg/kg)	2.64 ± 0.00^bc^	2.02 ± 0.00^c^	2.30 ± 0.00^c^	2.06 ± 0.70^ab^	5.28 ± 0.10^c^	1.40 ± 0.00^a^	2.12 ± 0.70^c^

*Note:* *n* = 6. Values are presented as mean ± SD. ^a–c^Values of the same column with different superscripts are significantly different at *p* < 0.05.

#### 3.4.4 Effect of Oils on NO Levels in Organ Homogenate and Serum

The concentrations of the NO in organ homogenate and sera of rat are presented in Table 8. The normal group, the group treated with 100% WO, 50:50 (GO:WO) and 60:40 (GO:WO) presented significantly (*p* < 0.05) higher NO levels compared with the negative control group and the groups treated with orlistat and 100% GO. The NO concentration in the kidney was significantly (*p* < 0.05) higher in the group treated with orlistat and 50:50 (GO:WO) 1000 mg/kg compared with the other groups. At the level of the heart, the group treated with 100% GO presented the highest (*p* < 0.05) NO level compared with the other groups. No significant (*p* < 0.05) difference was recorded between the NO levels of the negative control and the groups treated with orlistat, 100% WO, 50:50 (GO:WO) and 60:40 (GO:WO). At the level of the brain, the rats that received orlistat and 100% WO presented the highest (*p* < 0.05) NO levels compared with the other groups, the lowest value observed in the negative control group. The pancreas homogenate showed significantly (*p* < 0.05) higher NO levels in the normal group, followed by the groups treated with 50:50 (GO:WO), 100% WO, and orlistat compared with the other groups where the concentrations were significantly (*p* < 0.05) lower. At the level of the liver, the normal and the negative control groups presented similar (*p* < 0.05) NO levels which were significantly (*p* < 0.05) lower than those of the other groups. Looking at the NO levels of the sera, the normal group presented the lowest (*p* < 0.05) NO concentration, while the highest value was recorded in the serum of the rats treated with 100% WO.

**TABLE 8:** Effect of African walnut oil, groundnut oil, and their corresponding blends on NO levels in organ homogenate and serum (μM/L).

	Spleen	Kidney	Heart	Brain	Pancreas	Liver	Serum
Normal	16.84 ± 5.30^ac^	11.11 ± 0.00^a^	13.72 ± 3.07^a^	10.81 ± 0.00^ac^	37.73 ± 3.16^a^	28.34 ± 0.69^a^	1.31 ± 0.00^a^
Negative control	8.46 ± 1.41^b^	12.27 ± 0.00^a^	8.94 ± 3.63^b^	7.40 ± 0.41^b^	13.46 ± 0.57^b^	22.45 ± 3.22^a^	3.15 ± 1.55^b^
Orlistat (10 mg/kg)	10.27 ± 3.29^a^	34.31 ± 2.41^b^	9.59 ± 2.75^b^	12.58 ± 1.57^c^	22.05 ± 1.67^c^	36.15 ± 0.75^bc^	6.97 ± 0.91^c^
100% WO (1000 mg/kg)	15.98 ± 2.51^ac^	9.65 ± 1.05^a^	6.60 ± 2.62^b^	18.84 ± 1.19^d^	29.03 ± 0.00^d^	35.53 ± 2.63^bc^	11.24 ± 4.18^c^
100% GO (1000 mg/kg)	9.67 ± 0^b^	10.54 ± 3.70^a^	32.12 ± 2.07^c^	10.92 ± 0.12^ac^	18.74 ± 0.76^e^	37.76 ± 4.07^bc^	5.08 ± 0^d^
50:50 (GO: WO) (1000 mg/kg)	17.08 ± 1.43^c^	16.64 ± 0.55^c^	7.97 ± 0.94^b^	9.06 ± 2.20^ac^	31.74 ± 3.32^a^	42.11 ± 3.09^c^	4.94 ± 1.62^c^
60:40 (GO: WO) (1000 mg/kg)	14.32 ± 0^ac^	7.37 ± 0.19^a^	11.83 ± 1.66^b^	10.35 ± 3.90^ac^	10.63 ± 1.39^b^	33.89 ± 3.68^bc^	4.41 ± 3.45^c^

*Note:* *n* = 6. Values are presented as mean ± SD. ^a–d^Values of the same column with different superscripts are significantly different at *p* < 0.05.

## 4 Discussion

### 4.1 Initial Oil Quality

In our previous recent study, the oils used in this study (African walnut oil, GO, and their blends) were reported to have good quality parameters as recommended by the norm (Table 3) (Douky et al. 2025). The peroxide value, which is an indicator of the primary oxidation state of the oil, was found to range between 2.52 and 4.94 meq O_2_/kg (Douky et al. 2025) which is lower than 15 meq O_2_/kg as recommended by the norm for crude or cold pressed vegetable oils (WHO/FAO 1999). The *p*‐anisidine value, which measures secondary oxidation products, was found to fall between 7.07 and 12.33 (Douky et al. 2025) which was lower than the standard value of 20 as indicated for fish oil (WHO/FAO 2017). The authors (Douky et al. 2025) justified the use of fish oil parameters as standard by the fact that the *p*‐anisidine value boundaries are not defined yet for virgin and refined vegetable oils (Agriculture and Consumer Protection Department 1999). Similarly, Douky et al. 2025 showed that the TOTOX values of African walnut oil, GO, and their 50:50 and 60:40 blends were all found to be below 26, which is the mandated limit as reported by the GOED Voluntary Monograph, Version 5 (2015). For the acid value, it informs on the degree of hydrolysis of triglycerides in the oil, and Douky et al. (2025) found that they were generally higher than 0–4 mg KOH/g, which is the recommended range for crude and virgin vegetable oils (Agriculture and Consumer Protection Department 1999; FAO and WHO 1999). Douky et al. (2025) also reported iodine values of 86.41, 166.41, 112.12, and 93.21 Gi2/100 g, respectively, for GO, African walnut oil, GO:WO (50:50), and GO:WO (60:40).

The Fourier‐transformed infrared spectrum of the same oil samples was also done in our previous work, and the result is presented in Figure 1 (Douky et al. 2025). The report of these authors revealed the absence of peaks in all graphs within the range of 3250 to 3750 cm^−1^, which they explained could be due to the absence of O‐H stretching vibrations (hydroxyl (‐OH) functional groups) typically associated with alcohols and hydroperoxides. They also found peaks between 3000 and 2900 cm^−1^, which they attributed to the C‐H stretching vibrations of molecules in the oil. In the same line, these authors revealed an abundant peak at 1750 cm^−1^ in all figures, which they attributed to the stretching vibration of the carbonyl (C=O) functional group, commonly found in esters, aldehydes, and ketones. The authors also mentioned that the absence of peaks at 2750 cm^−1^ and 1600 cm^−1^ could be due to the absence of C‐H stretching vibrations of aldehydes and the C=C stretching vibrations in aromatic rings, respectively, confirming that the oils' qualities were good. At the level of the fingerprint region, peaks were obtained at 900 and 750 cm^−1^, which Douky et al. (2025) attributed to the strong bending vibration of C‐H bonds in alkenes or aromatic compounds. Generally, from the data published in our recently published study Douky et al. (2025), the oil characteristics were good.

### 4.2 Hematology

For the hematology results, low PLT values were observed in the group that received 100% GO, which could be due to a possible enhanced immune response or oxidative stress, potentially triggered by the HFD. Similar low HCT and LYM values were also observed with the HFD groups as compared with the normal group. Hematology is the study of blood, including its components and disorders. The hematology results of this study correlate with various research that indicated that HFD supplemented with certain edible oils could result in low HCT, PLT, and LYM counts. This reduction could, however, be associated with altered blood viscosity and impaired erythropoiesis due to the dietary fats influencing bone marrow function and overall production (Tai et al. 2010). High GRAN and MID values of groups that received 100% GO and 60:40 (GO:WO) could indicate a possible infection and possible immune suppression and infection. Various studies have reported that the consumption of different edible oils by HFD rats led to an increase in GRAN and MID counts. The studies suggested that this elevation could be linked to inflammatory responses triggered by the high fat content of the diet and the specific fatty acid profiles of the oils used (Altberg et al. 2020). A similar study was conducted by Tagauov et al. (2023) who carried out a study on the comparative effects of different supplemented dietary doses of chlorophyll on blood parameters of experimental rats and obtained the same results.

### 4.3 Inflammatory Cytokines

At the level of the cytokine, the negative control group exhibited the highest levels of cytokine compared with other groups. Inflammatory cytokines are signaling molecules that are produced by immune cells and various other cell types in response to inflammation, infection, or injury, and it is a significant factor in obesity‐related complications. The high inflammatory cytokine levels observed in the negative control group could be due to the effect of HFD on adipocyte hypertrophy of the rats, which gradually increased the levels of pro‐and anti‐inflammatory markers in them. This is in line with a study conducted by Albrahim et al. (2022) on the impact of dietary consumption of palm oil and olive oil on lipid profile and hepatocyte injury in hypercholesterolemic rats. The results of this study revealed that the negative control group fed a high‐cholesterol diet, exhibited a notable increase in pro‐inflammatory cytokines such as IL‐1β and TNF‐α. However, the lower cytokine levels in the treatment groups may suggest an anti‐inflammatory effect of the dietary interventions. Previous studies have shown that dietary patterns rich in anti‐inflammatory components can reduce cytokine levels, thereby mitigating the effects of oxidative stress and inflammation (Giugliano et al. 2006). The correlation between elevated NO levels and increased pro‐inflammatory cytokines observed in other studies further supports the notion that oxidative stress and inflammation are interconnected processes (Karbach et al. 2014).

### 4.4 Oxidative Stress

Studies showed that oxidative stress significantly contributes to the development of obesity by promoting the accumulation of fat in adipose tissues (Higuchi et al. 2013). Results showed that the rats that received the HFD exhibited lower levels of catalase (CAT) in the pancreas compared with the normal group, high levels of TBA value, and low NO levels as compared with the normal group. This suggests that the HFD group may have experienced oxidative stress and possible impaired antioxidant defenses (low CAT, high TBA), while also exhibiting compromised vascular function (low NO). This is consistent with the understanding of the negative effects of HFD and obesity on metabolic and cardiovascular health. On the other hand, the normal group demonstrated better oxidative balance and vascular health. This is consistent with the study conducted by Lasker et al. (2019) who also observed similar results in a study on the effects of HFD‐induced metabolic syndrome and oxidative stress in obese rats ameliorated by yoghurt supplementation. The results of this study showed that the rats on HFD exhibited high concentrations of MDA, and decreased activities of antioxidant enzymes like CAT compared with the control groups which were as a result of antioxidant defenses that were compromised in rats taking the HFD. The same study noted that the rats that received the HFD exhibited increased oxidative stress parameters, which correlated with the observed decrease in NO levels, further indicating compromised vascular function.

Similarly, another study conducted by Kangani et al. (2022) suggested the link between multisource edible oils in reducing cholesterol levels and improving cardiovascular health due to its balanced composition of MUFA and PUFA. The multisource edible oils and their blends used in the present study exhibited high GSH levels in the brain homogenate of rats that received the 60:40 (GO:WO) blend; in the pancreas homogenate of the group that received 100% GO, and the serum of the rats that received 50:50 (GO:WO). Low GSH activity was also exhibited in the kidney homogenate of rats that received 100% GO, and liver homogenate for the rats that received 50:50 (GO:WO). GSH is an antioxidant that helps reduce oxidative stress. The results obtained from the GSH activity may suggest that the 100% GO had a negative effect on the GSH activity. However, the 60:40 (GO:WO) blend may have a protective effect on GSH activity in the brain and the 50:50 (GO:WO) blend having a protective effect in the serum.

NO levels were shown to be higher in the normal group, the groups that received 100% WO, 50:50 (GO:WO) and 60:40 (GO:WO). High NO levels were also seen in the kidney homogenate of groups that received 50:50 (GO:WO) and orlistat respectively; in the heart homogenate of the group that took 100% GO; in the brain homogenate of the groups that received orlistat and 100% WO in the pancreas homogenate for the normal group, followed by the groups that received 50:50 (GO:WO), 100% WO, and orlistat; and in the serum for the group that took the 100% WO group. NO is a signaling molecule involved in various physiological processes including vasodilation and metabolic regulation (Pourbagher‐Shahri et al. 2021). The significant increase in NO levels observed in the normal and treated groups compared with the negative control suggests that the oils may possess properties that stimulate NO synthesis. This is consistent with previous studies indicating that certain dietary oils can influence endothelial function and NO production. For example, a study indicated that olive oil consumption positively correlates with increased NO levels due to its high content of polyunsaturated fatty acids, which promote endothelial function and vasodilation (Caner et al. 2005). Similarly, the variability in NO levels across different organs (kidney, heart, brain, pancreas, and liver) highlights the organ‐specific effects of the treatments. For instance, the kidney showed significantly higher NO levels in the groups taking orlistat and 50:50 (GO:WO), which may indicate a protective or adaptive response to the treatment. In contrast, the heart exhibited the highest NO levels in the group that received 100% GO, suggesting that this oil may have cardioprotective effects. The liver and pancreas results are particularly noteworthy. The similar NO levels in the normal and negative control groups suggest a lack of stimulation in these organs under the conditions tested. This could imply that the oils may have a limited effect on NO production in the liver and pancreas compared with other organs.

Catalase is an enzyme that catalyzes the decomposition of hydrogen peroxide (H_2_O_2_) into water and oxygen, thereby protecting the cells from oxidative damage (Rasheed 2024). The high CAT levels in the liver of the groups that received the 50:50 (GO:WO) blend may be due to a protective effect against oxidative stress in the liver, which has been shown to be associated with non‐alcoholic fatty liver disease (Na et al. 2019) while the low CAT for the group treated with 100% WO could be an indicator of possible oxidative stress in the blood. Alternatively, the low TBA levels observed in the group that received the normal diet, the HFD plus 100% WO, and the negative control group for the kidney and liver may indicate a decrease in lipid peroxidation, which is a process where free radicals attack lipids in the cell membranes, causing cellular damage. The reduction of TBA levels suggests that the oils may protect cellular membranes from oxidative damage, leading to healthier organ function. These results are in line with a study conducted by Uti et al. (2020) who showed that African walnut attenuates ectopic fat accumulation and associated peroxidation and oxidative stress in monosodium glutamate–induced obese Wistar rats. The results of this study showed that the supplementation of African walnut oil in obese rat models led to an increased level in GSH and CAT while simultaneously decreasing TBA levels, indicating reduced lipid peroxidation and improved antioxidant status in the liver and other tissues. Another study carried out by Ramesh et al. (2006) also evaluated the effect of dietary substitution of GO on blood glucose, lipid profile, and redox status in streptozotocin‐induced diabetic rats, showing that GO could significantly elevate GSH and CAT levels alongside reduced TBA levels in diabetic and obese rat models. The rats in this study were given an HFD with GO.

## 5 Conclusion

Results showed that oil quality indices were within normal ranges, and the blends presented similar colors. Organ weights and oxidative stress parameters showed significant differences, with improved catalase, GSH, and NO levels in oil‐treated groups. Hematological parameters remained unchanged, while serum inflammatory cytokines were significantly reduced in oil‐treated groups. Hence, it can be concluded that GO, African walnut oil, and their blends have a role in protecting against obesity and its effects on oxidative stress, hematology, and inflammatory cytokines. GO, African walnut oil, and their blends can be recommended in the diet of obese patients to improve obesity and its comorbidities. The production and consumption of these oil blends should be encouraged, particularly in regions where obesity is prevalent pending toxicological studies.

## Author Contributions

**Thelma Besong Taku:** conceptualization (equal), data curation (equal), investigation (equal), methodology (equal), resources (equal), writing – original draft (equal), writing – review and editing (equal). **Fabrice Tonfack Djikeng:** conceptualization (equal), data curation (equal), formal analysis (equal), investigation (equal), methodology (equal), resources (equal), supervision (equal), validation (equal), visualization (equal), writing – original draft (equal), writing – review and editing (equal). **Bertrand Ayuk Tambe:** conceptualization (equal), data curation (equal), investigation (equal), methodology (equal), resources (equal), validation (equal), visualization (equal), writing – original draft (equal), writing – review and editing (equal). **Veshe‐Teh Zemoh Sylvia Ninying:** methodology (equal), writing – original draft (equal), writing – review and editing (equal). **Evans Mainsah Ngandung:** conceptualization (equal), data curation (equal), methodology (equal), resources (equal), validation (equal), visualization (equal), writing – original draft (equal), writing – review and editing (equal).

## Ethics Statement

This is to inform you that in this study, animals were involved. Animals were cared for and used in agreement with international standard guidelines for animal use. To carry out this study, an ethical clearance for animal handling and care was obtained from the University of Buea—Institutional Animal Care and Use Committee (IACUC) with permit UB‐IACUC No 02/2024.

## Conflicts of Interest

The authors declare no conflicts of interest.

## Data Availability

The data used and/or analyzed during the current study are available from the corresponding author upon reasonable request.
